# Right subclavian double steal syndrome: a case report

**DOI:** 10.1186/1752-1947-2-392

**Published:** 2008-12-23

**Authors:** Konstantinos Filis, Levon Toufektzian, Frangiska Sigala, Dimitrios Kardoulas, Aikaterini Kotzadimitriou, Emmanuel Lagoudianakis, Nikolaos Koronakis, Andreas Manouras

**Affiliations:** 1 Department of Propaedeutic Surgery, Hippokrateion Hospital, Athens Medical School, University of Athens, Athens, Greece; 2 Second Department of Surgery, 417NIMTS – Nosileutiko Idrima Metohikou Tameiou Stratou (Military Veterans' Fund Hospital), Athens, Greece

## Abstract

**Introduction:**

Double-steal syndrome represents a causative factor for blood flow compromise of the cerebral vascular bed with transient neurologic symptoms. We present the case of a patient with innominate artery atherosclerotic occlusion, manifested as blood flow reversal in the vertebral and common carotid arteries. Symptomatic atherosclerotic occlusive disease of the innominate artery is relatively rare and represents less than 2% of all extracranial causes of cerebrovascular insufficiency.

**Case presentation:**

We report on a 73-year-old male patient who presented at our hospital for the evaluation of dizziness and episodes of syncope. Angiography and color Doppler examinations documented the double syndrome as retrograde flow in the right vertebral artery and the right carotid artery.

**Conclusion:**

Constituting an indication for surgical correction, his condition was managed with the performance of carotid-carotid extra-anatomic bypass for the permanent reestablishment of antegrade blood flow in the vascular network supplying the brain. Carotid-carotid extra-anatomic bypass was a good option for our patient, since he remains symptom free after one year of follow up.

## Introduction

Symptomatic atherosclerotic occlusive disease of the innominate artery is a relatively infrequent condition when compared with other manifestations of atherosclerosis causing flow-limiting effects and comprising less than 2% of all extracranial causes of cerebrovascular insufficiency [1]. Inflow obstruction of the right subclavian and common carotid arteries poses significant risks since right cerebral and right upper limb arterial blood supply depends on collateral networks which may not suffice in the presence of increased demand, promoting the appearance of neurologic deficits of varying degrees. Clinical presentation ranges from asymptomatic stenosis to more ominous forms of vascular compromise and subclavian steal syndrome manifesting with transient cerebral ischemia and ischemic arm symptoms. Although it was initially believed that the presence of subclavian stenotic or occlusive disease was associated with cerebral ischemia and neurologic deficits related to vertebrobasilar hypoperfusion, this opinion has been challenged and the prerequisite for the development of symptoms is the presence of disease in other extracranial vessels supplying the brain [2]. This observation is due to the relatively small contribution of the vertebral arteries to cerebral blood flow; a fact that has been demonstrated experimentally with no change in cerebral blood flow or electroencephalogram (EEG) activity in the presence of reversed vertebral artery flow [3]. Patients with aortic branch occlusive disease represent a population at high risk [4] for the development of hemodynamic and embolic complications. The presence of innominate artery occlusion proximal to the origin of the common carotid and vertebral arteries may result in retrograde blood flow in one or both of these vessels and preferential blood flow into the low pressure ipsilateral upper limb vessels during exercise or even rest, depending on the presence of collateral flow and on the relative resistance of the vascular beds. This phenomenon can be described as subclavian-carotid double steal syndrome.

## Case presentation

**Figure 1:**
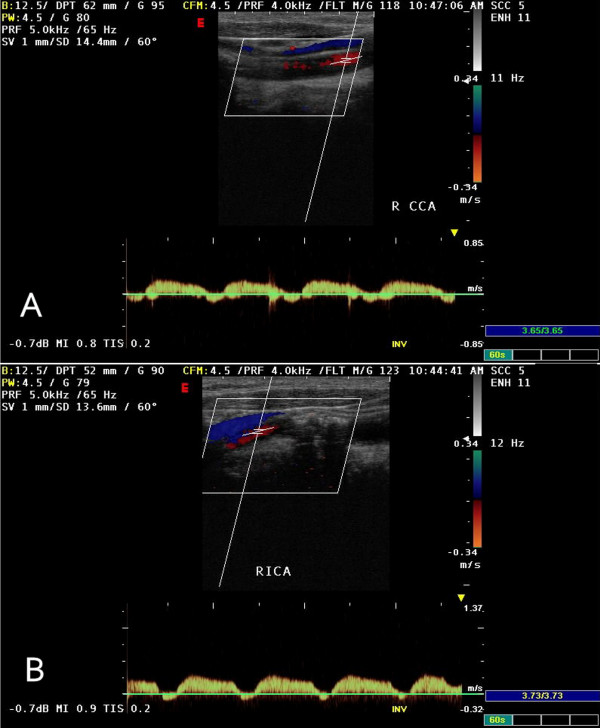
**Duplex ultrasonography of the right common carotid artery depicting the biphasic antegrade blood flow during cardiac systole and retrograde blood flow towards the right subclavian artery during cardiac diastole (subclavian-carotid steal syndrome)**.

**Figure 2:**
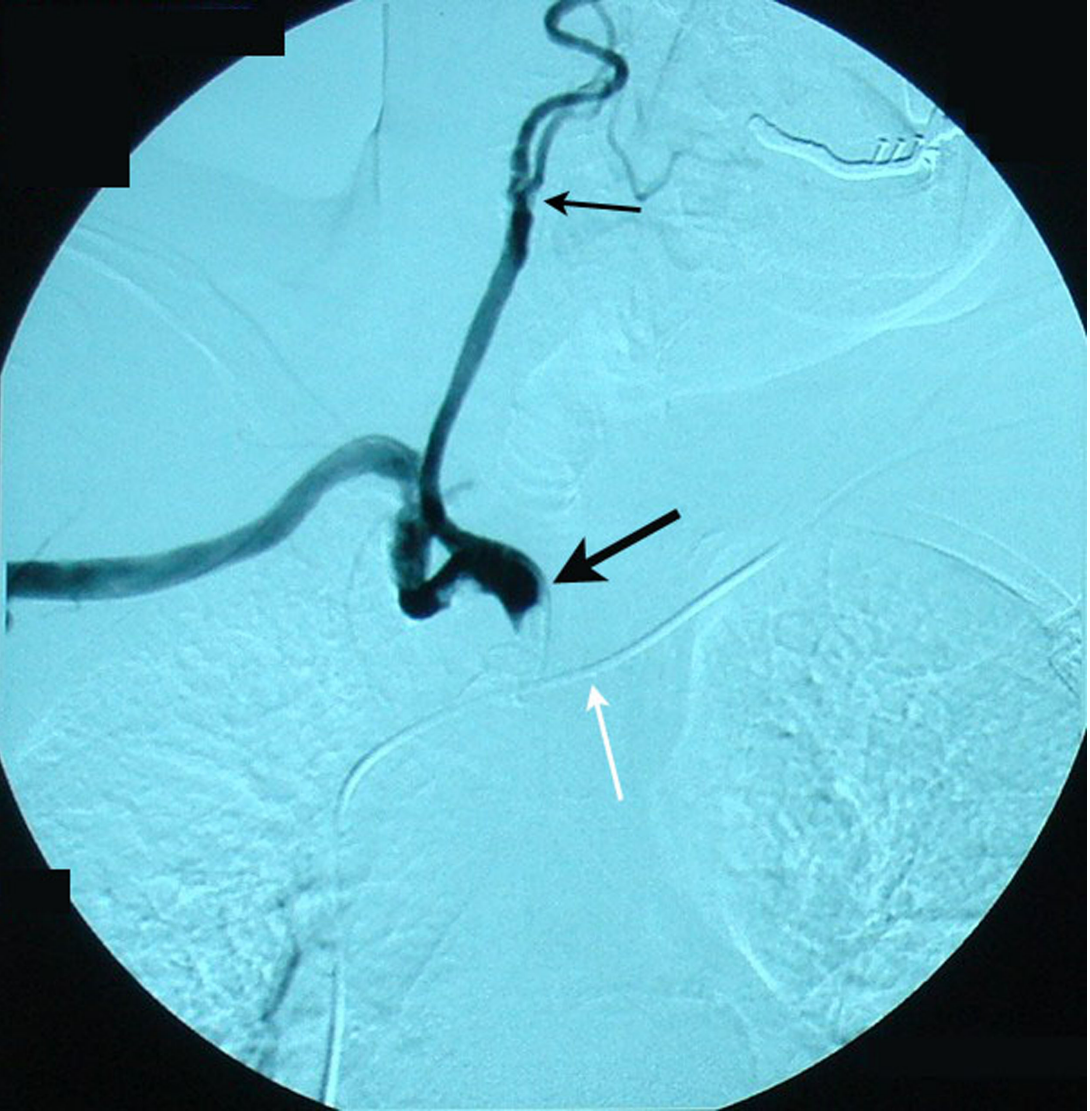
**Angiography after catheterization of the innominate artery near the origin of the right common carotid and subclavian arteries demonstrating the occlusion of the brachiocephalic trunk (bold black arrow)**. Note that contrast media is not running along the right vertebral artery due to the retrograde blood flow in the vessel (subclavian-vertebral steal syndrome). Concomitant stenosis of the common carotid artery (thin black arrow) is a manifestation of the generalized atherosclerotic disease. Stenotic lesions of initial and mid-portion segments in the subclavian artery were not considered to be that significant for acute treatment. In case they progressed we considered endovascular treatment. White arrow: pacemaker wire.

**Figure 3:**
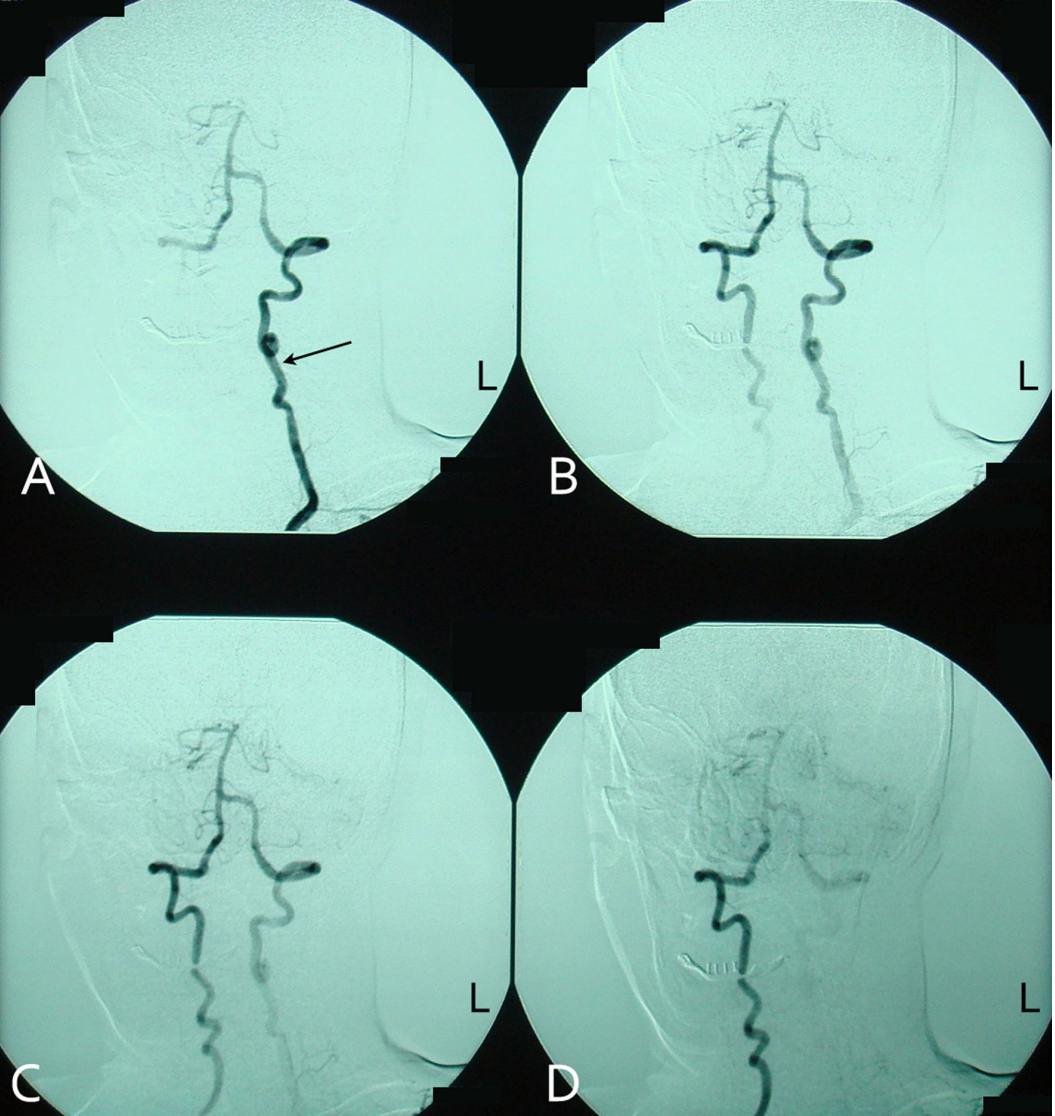
**Subsequent angiographic images of the vertebrobasilar arterial network after catheterization of the left subclavian artery and contrast media instillation**. Blood flows through the left vertebral artery (A), partly supplies the basilar artery (B and C), but preferentially runs along through the right vertebral artery towards the right subclavian artery (B, C and D) depicting the subclavian-vertebral steal syndrome. Arrow: left vertebral artery.

A 73-year-old man with diabetes mellitus, hypertension and hypercholesterolemia, and a previous history of coronary artery bypass surgery presented for evaluation of dizziness and episodes of syncope. Bilateral cervical bruits, a difference in blood pressure 45–50 mmHg between both arms, as well as barely discernable radial, ulnar and branchial arterial pulses on the right upper limb prompted color duplex which revealed bilateral internal carotid artery stenosis of 75% at each side, retrograde flow in his right vertebral artery (subclavian-vertebral steal) and retrograde flow in his right carotid artery during the midsystolic phase of the cardiac cycle (subclavian-carotid steal), while an antegrade blood flow was seen during the rest of the cardiac cycle (Figure 1). Further investigation with selective catheterization of the origin of the right subclavian artery and digital subtraction angiography demonstrated occlusion of the innominate artery (Figure 2) while catheterization of the left subclavian artery confirmed the subclavian-vertebral steal phenomenon on the right side (Figure 3). Due to bilateral external and common-external (on the left) iliac artery occlusions (the patient was a 200 meters claudicant), we had to perform upper extremity catheterization and full aortic arch aortography. Initially we performed a left brachial catheterization but we did not pass the aortic arch due to a dissecting plaque at the origin of the left subclavian artery (< 50%). We only performed selective catheterization of the left subclavian artery (Figure 3). Catheterization through the impalpable right brachial artery was the option for catheterization of the right subclavian artery, the wire passed the innominate lesion (possibly through) the subendothelial route. Clinical neurologic evaluation and brain computed tomography (CT) scan did not reveal focal ischemic brain lesions, while no myocardial ischemic symptoms and an ejection fraction of 40% was seen after regular post-coronary artery bypass grafting (using saphenous veins) (CABG) follow-up. We had to provide the most safe and effective clinical solution to this 73-year-old man with very extensive and severe arterial disease and a previous CABG. Permanent dizziness and many episodes of syncope with acceptable cardiac function was the indication for doing something invasively. The priorities for the patient were: a) reestablishment of antegrade arterial flow through the right carotid and right vertebral arteries; b) treatment of the bilateral internal carotid artery stenosis; c) treatment of the left subclavian dissecting lesion; and d) treatment of bilateral iliac artery occlusions.

The operation was conducted under general anesthesia and included a staged bilateral approach with a left cervical incision along the anterior border of the left sternocleidomastoid muscle employed to expose the left common carotid artery and to perform the endarterectomy with a Dacron patch angioplasty and the proximal end to side polytetrafluoroethylene (PTFE) graft-arterial anastomosis with the use of a shunt, while a limited right cervical incision was used to expose the origin of the right common carotid artery and to perform the peripheral end to side graft-arterial anastomosis passing anteriorly to the trachea under the staple muscles. We preferred left carotid to right carotid bypass instead of left subclavian to right carotid bypass, to avoid double length of graft and dysfunction in neck movements, besides in our case left subclavian had an ostial < 50% stenosis. Concerning the internal carotid stenosis, we treated the left one since it was more ulcered and our policy is to treat the left lesion first and later the right one, after close follow-up. When performing treatment of the right internal carotid stenosis we tried an effective solution to its coiling. Endovascular treatment of the innominate occlusion was an option with specific disadvantages: a) the lesion could possibly be passed with a wire but then the angioplasty would be performed rather in a subendothelial route or, in an extremely eccentric way in a vessel which is known to be fragile and the patient having had a previous sternotomy, rendering the emergent operation in case of rupture, a catastrophe; b) the possibility of having an alternative second endovascular approach (second wire) through femoral or left subclavian arteries was deemed neither efficient nor safe; c) the feasibility of innominate endovascular angioplasty is known to have a high risk of distal embolization. In this case the operator had to put one or two filters for protection of embolization (carotid and subclavian); d) the insertion of a filter in the right common carotid, at least, was technically very difficult or unfeasible before innominate angioplasty, as the only route was the right subclavian and the angioplasty had to be done in a retrograde way in the innominate artery; and e) the medium and long term results of subclavian angioplasty are inferior to operative ones, especially for innominate occlusions or sub-occlusions and we are unaware of a series with medium or long term results.

We have an excellent series of 0% death-stroke rate of carotid endarterectomies (70 cases) and we have not yet adopted the questionable carotid angioplasty combined with stenting (CAS). The patient remains symptom free after one year of follow-up, while the right internal carotid artery stenosis remains stable.

## Discussion

Obstructive lesions of the innominate artery occur more proximally than those leading to subclavian steal syndrome. The resulting inflow compromise of both subclavian and common carotid arteries is responsible for the higher incidence of symptomatic disease among patients with innominate artery lesions. The spectrum of manifestations ranges from upper extremity exercise-induced ischemia to cerebrovascular insufficiency. Obstruction of the innominate artery not affecting the origin of the subclavian and common carotid arteries allows free communication between these vessels, which receive a substantial part of their arterial supply from collateral circulation. The combination of disturbed arterial inflow, along with differences between vascular resistances of the cerebral and upper extremity vascular beds, is responsible for a complex blood flow relationship giving birth to partly or totally abnormal vascular flow directions in the involved vessels. Since, during innominate artery occlusion, right sided cerebral blood flow is achieved by means of collateral routes involving the circle of Willis, and diversion of flow across the vertebrobasilar junction is more effective than across the circle of Willis [5], a permanent retrograde blood flow in the right vertebral artery (Figure 3) and a transient mid-systolic blood flow reversal in the common and internal carotid arteries (Figure 1) are observed. A recent report showed that complete occlusion of the right vertebral artery was the cause for the so called innominate disease phenomenon [5].

Quantification of steal from the vertebral and carotid arteries is not possible, since it is dependent on the complex pattern of collateral routes developed due to innominate artery occlusion. These lesions occur progressively, triggering the genesis of complex collateral arterial networks for both right sided cerebral and right upper extremity supply. Additionally, blood flow is readily affected from peripheral resistances of the upper extremity and cerebral arterial networks. It is well known that right upper extremity exercise causes arterial vasodilation, reduction of resistance and increased steal from both vertebral and right carotid arteries. However, the contribution of the carotid system to upper extremity arterial inflow is affected by stenoses of the common and internal carotid arteries as well.

Restoration of this complex flow relationship needs a thorough understanding of blood flow physics in integration with relative vascular beds resistance and knowledge of collateral arterial network development. Symptomatic reversal of flow of the right vertebral artery is associated with innominate artery disease in only 10% of cases [6]. The diagnosis of significant stenosis or occlusion of the innominate artery is based on sonographic findings of right vertebral artery retrograde flow along with reversal of flow of the common and internal carotid arteries. Transient flow reversal at the common carotid indicates disturbed inflow of this artery. Selective transluminal arteriography of the right subclavian artery and of the vertebrobasilar system anatomically confirms occlusion of the innominate artery with intact origins of the right subclavian and common carotid arteries. The decision for surgical repair should be based on clinical grounds, as are overt ischemic symptoms. Symptomatic occlusive disease of the innominate artery is relatively rare, so we have little or no knowledge on medium or long term results of the endovascular or operative approach. Concerning the subclavian occlusive lesions, medium and long term results of subclavian angioplasty are inferior to the operative ones. The clinical manifestations indicated surgical treatment, since cardiac function was acceptable. The endovascular approach was considered neither safe nor possible. The lesion could be possibly passed with a wire but then the angioplasty would be performed rather in a subendothelial route or in an eccentric way, a second endovascular approach through femorals or subclavian would be difficult, there was risk of distal embolization and insertion of a filter in the right common carotid artery before angioplasty would be almost impossible before angioplasty.

## Conclusion

The goal of surgical intervention is the restoration of permanent and stable antegrade blood flow in the extracranial vessels with altered blood flow on noninvasive and invasive modalities, as described above, correcting cerebral hypoperfusion and its associated symptoms. At the same time, care must be taken to avoid lengthy surgical procedures with high associated morbidity and mortality, taking into consideration the high risk status of these patients. The choice of an extra-anatomic bypass procedure gave good results in terms of morbidity and mortality, patency rates and technical simplicity [7]. Carotid-carotid bypass with the performance of the distal anastomosis in close proximity to the origin of the right common carotid artery constitutes the option of choice supplying blood flow to the right carotid artery system, as well as to the right subclavian-vertebral arteries system. A theoretic concern with use of an artery as a donor is the possibility of diminished flow to its own distal vascular bed. However, this concern has proven to be inaccurate, by clinical studies, which have demonstrated that a subclavian or a carotid artery free of proximal stenotic disease can be used as an inflow source for other major branch systems [8], without the threat of steal and with greatly increased blood flow supplying more than one distal vascular bed [9]. Endovascular techniques have recently been performed with favorable results for patients with aortic arch stenotic disease [10]. However, in the presence of occlusive disease, the efficacy and long term patency of this approach is questionable.

## Abbreviations

EEG: electroencephalogram; PTFE: polytetrafluoroethylene; CAS: carotid angioplasty combined with stenting; CT: computed tomography; CABG: coronary artery bypass graft

## Competing interests

The authors declare that they have no competing interests.

## Authors' contributions

KF contributed to final approval, revision and drafting of the manuscript. LT contributed to literacy search and drafting of the manuscript. FS contributed to final approval, revision and drafting of the manuscript. DK contributed to data collection, interpretation of data and literature search for the manuscript. AK contributed to literacy search and drafting of the manuscript. EL contributed to data collection, interpretation of data and literature search of the manuscript. NK contributed to data collection, interpretation of data and literature search of the manuscript. AM contributed to final approval, revision and drafting of the manuscript. All authors read and approved the final manuscript.

## Consent

Written informed consent was obtained from the patient for publication of this case report and accompanying images. A copy of the written consent is available for review by the Editor-in-Chief of this journal.

## References

[B1] Wylie EJ Effeney DJ Surgery of the aortic arch branches and vertebral arteries Surg Clin North Am 1979 59 669 680 382406 10.1016/s0039-6109(16)41887-6

[B2] Moran KT Zide RS Persson AV Jewell ER Natural history of subclavian steal syndrome Am Surg 1988 54 643 644 3189998

[B3] Eklof B Schwartz SI Effects of subclavian steal and compromised cephalic blood flow on cerebral circulation Surg Forum 1969 20 154 156 4910574

[B4] Kessler C Mitusch R Guo Y Rosenqart A Sheikhzaden A Embolism from the aortic arch in patients with cerebral ischemia Thromb Res 1996 84 145 155 10.1016/0049-3848(96)00171-5 8914214

[B5] Grant EG El-Saden SM Madrazo BL Baker JD Kliewer MA Innominate artery occlusive disease: sonographic findings AJR Am J Roentgenol 2006 186 394 400 10.2214/AJR.04.1000 16423944

[B6] Killen DA Gobbel WG Jr Subclavian steal-carotid recovery phenomenon J Thorac Cardiovasc Surg 1965 50 421 426 14346549

[B7] Perbellini A Lievore R Mazzilli G Candiani P Ghini P Morando S Subclavian and innominate steal syndrome (our experience with 29 patients) Chir Ital 1987 39 338 345 3319235

[B8] Martin RS 3rd Edwards WH Muhlerin JLJR Edwards WH Jr Surgical treatment of common carotid artery occlusion Am J Surg 1993 165 302 306 10.1016/S0002-9610(05)80830-X 8447533

[B9] Abou-Zamzam AM Jr Moneta GL Edwards JM Yeager RA McConnell DB Taylor LM Jr Porter JM Extrathoracic arterial grafts performed for carotid artery occlusive disease not amenable to endarterectomy Arch Surg 1999 134 952 956 10.1001/archsurg.134.9.952 10487589

[B10] Sullivan TM Gray BH Bacharach JM Perl J 2nd Childs MB Modzelewski L Beven EG Angioplasty and primary stenting of the subclavian, innominate, and common carotid arteries in 83 patients J Vasc Surg 1998 28 1059 1065 10.1016/S0741-5214(98)70032-1 9845657

